# Genetic Aspects of Tooth Agenesis

**DOI:** 10.3390/genes16050582

**Published:** 2025-05-15

**Authors:** Clarissa Modafferi, Ilaria Tucci, Francesco Maria Bogliardi, Elena Gimondo, Pietro Chiurazzi, Elisabetta Tabolacci, Cristina Grippaudo

**Affiliations:** 1 UOC Genetica Medica, Fondazione Policlinico Universitario A. Gemelli IRCCS, 00168 Rome, Italy; clarissamodafferi@libero.it (C.M.); francesco.bogliardi01@icatt.it (F.M.B.); pietro.chiurazzi@unicatt.it (P.C.); 2 Dipartimento Universitario Testa Collo ed Organi di Senso, Università Cattolica del Sacro Cuore, 00168 Rome, Italy; ilaria.tucci01@gmail.com; 3 Department of Biomedical, University of Milan, Surgical and Dental Science, Unit of Orthodontic, Santi Paolo & Carlo Hospital, 20142 Milan, Italy; elena.gimondo@studenti.unimi.it; 4 Dipartimento di Scienze della Vita e Sanità Pubblica, Sezione di Medicina Genomica, Università Cattolica del Sacro Cuore, 00168 Rome, Italy; 5 UOC Clinica Odontoiatrica, Dipartimento di Neuroscienze, Organi di Senso e Torace, Fondazione Policlinico Universitario A. Gemelli IRCCS, 00168 Rome, Italy

**Keywords:** tooth agenesis, selective tooth agenesis, syndromic tooth agenesis, genetic basis of tooth agenesis, clinical classification

## Abstract

Tooth agenesis is among the most prevalent congenital anomalies affecting human dentition, characterized by the developmental absence of one or more teeth. This condition may be present in either syndromic or non-syndromic forms, with significant implications for oral function, aesthetics, and craniofacial development. This narrative review aims to provide a comprehensive overview of tooth agenesis, defining its classification, genetic underpinnings, epidemiological aspects, phenotypic features, and therapeutic approaches. Recent advances in genetic research have identified numerous causative genes, notably *EDA*, *MSX1*, *WNT10A*, and *PAX9*, each associated with specific patterns of missing teeth and involved in isolated and/or syndromic forms. Additionally, genes such as *TSPEAR*, *LRP6*, *PITX2*, and *GREM2* contribute to varying degrees of severity and tooth distribution, often blurring the lines between syndromic and isolated cases. The genotype-phenotype correlations underscore the complexity of the underlying molecular pathways involved in odontogenesis. From a therapeutic perspective, the management of tooth agenesis requires a multidisciplinary approach, often involving orthodontic, prosthetic, and surgical interventions tailored to the severity of tooth loss and patient age. Early diagnosis represents a crucial role in treatment planning, facilitating timely intervention during growth and enhancing long-term outcomes. In conclusion, tooth agenesis remains a complex clinical condition with a strong genetic basis. A patient-centered and interdisciplinary strategy is essential to address both functional and psychosocial needs.

## 1. Introduction

Human dentition is the result of a complicated process that begins during embryonic development and is completed only when facial development comes to an end. The need to extend this process for such a long period is due to the development of the teeth, starting from the dental crowns to the complete formation of the roots, always showing their final dimensions in every part. On the contrary, the jaws, where they are located, follow the growth process that provides for the progressive increase in size during the development period [1]. The process of tooth development and the mechanism of tooth eruption are so complex that dental anomalies are frequently observed. These can be classified into anomalies of number, shape, structure, and position. Dental anomalies have a genetic origin. However, there are still many obscure points about their formation.

Research is aimed at identifying factors that can influence tooth formation and often uses experiments on animals [2,3].

Anomalies due to the lack of number are called dental agenesis and are among the most frequent dental anomalies. The congenital absence of one or more teeth represents a clinical problem that still today deserves to be explored and studied in its various aspects. In some cases, a correlation between genetic alterations and dental agenesis has been demonstrated [4,5,6,7].

Dental agenesis may be associated with other malformations of the craniofacial skeleton. The study by Scribante and colleagues [8] analyzed the association between sella turcica bridging and some dental anomalies (impacted canines, the most common dental agenesis, and hyperdontia). Based on sella turcica dimensions, bridging was divided into three groups: group 1 without calcification, group 2 with partial calcification, and group 3 with complete calcification of the sella turcica. The results showed a statistical correlation between the sella turcica bridging and the presence of dental anomalies.

An interesting retrospective study analyzed the association between the presence of congenital dental agenesis and the so-called “Ponticulus Posticus”, which means the calcification of the Atlantooccipital Ligament (AOL) [9]. In particular, the lateral cephalometrics of 350 non-syndromic patients were analyzed in search of calcification of the AOL. Patients with tooth agenesis were compared with a control group without congenital dental agenesis. The results showed a positive correlation between the Ponticulus Posticus and dental anomalies, such as agenesis: In the group of patients with dental agenesis, the frequency of Ponticulus Posticus was reported to be 66.6%.

The evolution of knowledge and technologies entails the need to update the state of the art to have broader bases on which to base the research of etiopathogenetic causes and to design the most suitable therapies. For the most frequent agenesis, the scientific debate is alive and has led to the study of new therapeutic means to overcome functional and aesthetic problems. Sometimes dental agenesis is associated with other dental anomalies.

Frequently, the absence of an upper lateral incisor is observed, while the contralateral is microdontic, described as the presence of a single tooth smaller than average size [10]. The congenital absence of a tooth in the aesthetic area of the mouth offers the possibility of choosing different clinical scenarios, such as closing the space with mesialization of the canines, tooth-supported fixed prostheses such as Maryland-type prostheses, and dental implants [11]. The aesthetics can be further improved with the application of veneers [12].

In the rarest and most severe cases, however, knowledge is based on the description of isolated or familiar clinical cases, with a lower level of scientific evidence. The aim of this narrative review is to report the knowledge on dental agenesis from the clinics to the etiopathogenesis in order to allow the clinicians a more comprehensive diagnostic process providing personalized therapy. In the diagnostic field, the major innovations are related to the results of genetic research, which over the years have become increasingly accessible and useful for investigating rare diseases but with a less negative impact on the health of patients, such as tooth agenesis. In the therapeutic field, new orthodontic and prosthetic technologies have developed solutions that are attentive not only to function but also to the aesthetics of patients, achievable thanks to the new materials available in dentistry and the new diagnostic imaging methods that allow for more accurate planning of interventions.

## 2. Material and Methods

We referred to the SANRA scale for the quality assessment of narrative review articles [13]. Articles were selected based on specific keywords on PubMed (i.e., genetic basis of tooth agenesis, selective tooth agenesis, and syndromic tooth agenesis) and referred to single gene involved in tooth agenesis.

## 3. Classification

Most classifications of tooth agenesis are based on the number of congenitally missing teeth.

One of the oldest and most recognized classifications was that proposed in 1953 by Malavez [14], who was appreciated for his schematic and simplified approach. This classification distinguishes between the following:

- Anodontia, which can be either total anodontia, meaning the complete absence of teeth, or complete anodontia, referring to the absence of teeth in only one dentition. The latter is further divided into agenodontia, defined as the absence of primary dentition, and ablastodontia, indicating the absence of permanent dentition;
- Oligodontia, referring to the absence of at least half of the expected number of teeth. This condition is subclassified as oligogenodontia, characterized by the presence of 10 or fewer primary teeth, and oligoblastodontia, defined by the presence of 16 or fewer permanent teeth;
- Hypodontia, the absence of fewer than half of the normal dental complement. This includes atelegenodontia (more than 10 but fewer than 20 primary teeth) and ateleblastodontia (more than 16 but fewer than 32 permanent teeth).

Subsequent studies have defined hypodontia as the absence of fewer than six teeth [15,16,17,18], excluding third molars. Anodontia has been further described as the failure in the development of all teeth [17,18,19,20]. Oligodontia is defined as the congenital absence of six or more permanent teeth [17,18,19]. Hypodontia can be further classified as non-syndromic (isolated) or syndromic when associated with systemic conditions or syndromes [21]. The non-syndromic form is the most common presentation of hypodontia [22]. One of the studies analyzing dental phenotypes in syndromic versus non-syndromic cases is the one by Dhamo and colleagues [23], where 129 patients with isolated oligodontia and 22 with ectodermal dysplasia (ED)-associated oligodontia were analyzed. The ED group exhibited more severely disrupted dental development, with abnormally shaped incisors and canines, a higher frequency of missing maxillary and mandibular central incisors and second molars, and mandibular lateral incisors. Many syndromic conditions are associated with oligodontia, such as Down syndrome and Ellis van Creveld syndrome [24]. Regarding mesiodistal angulation of unerupted mandibular second premolars, a greater distal inclination was noted in the oligodontia group compared to controls [25].

Congenital malformations have been correlated with tooth agenesis, such as an oral cleft lip and/or palate [26].

Over the years, increasing research has explored potential correlations between agenesis and various phenotypic, facial, and occlusal features. An important systematic review by Rakhshan and colleagues [27] aimed at determining the mean number of missing teeth (NMT) per individual and identifying influencing factors. Results showed an average of 1.5 NMT among randomly selected subjects and 2 NMT in dental/orthodontic patients. The number was not significantly influenced by region, ethnicity, or time period.

In efforts to identify specific agenesis patterns across populations, Arai [25] studied panoramic radiographs of 228 Japanese orthodontic patients with non-syndromic oligodontia (excluding third molars). The highest frequency of missing teeth was found in the maxillary and mandibular second premolars, followed by the maxillary first premolars.

The association between hypodontia and temporomandibular disorders (TMDs) or malocclusions has also been investigated. An analysis of pre-treatment records of 601 patients in Nepal revealed that although hypodontia was more commonly observed in Class II malocclusions, followed by Class I and Class III, no statistically significant difference among the classes was found [28]. The most frequently missing tooth was the maxillary lateral incisor, followed by mandibular lateral and central incisors, and mandibular second premolars—excluding third molars.

The absence of teeth may influence several occlusal and metrical characteristics. A study by Alamoudi and colleagues [29] found that a greater number of missing teeth was associated with reduced overjet, increased interincisal angle, decreased maxillary and mandibular arch lengths, and reduced labial inclination of anterior teeth. Missing posterior teeth in particular may contribute to TMD development. In a study by Wang and colleagues [30] involving 741 patients, those with posterior tooth loss in multiple quadrants—especially young females—were more likely to develop TMDs. A comparison between individuals with congenitally missing teeth and controls showed a significantly higher prevalence of TMD in the former group [31].

## 4. Epidemiological Data

The frequency of tooth agenesis varies in relation to the number and position of the missing teeth, the geographical area of origin, and sex [32].

The teeth most commonly affected by agenesis are third molars, with a prevalence of 22.6%, with Asian populations showing the highest rate of 29.7% [33]. Even in the case of the frequency of third molar agenesis, epidemiological studies report a significant increase in individuals also affected by other dental agenesis [34].

Tooth agenesis is very rare in deciduous dentition, and the prevalence ranges between 0.1% and 0.2% [34,35], with similar values reported among Caucasian populations and in New Zealand, whereas higher incidence rates have been observed in Japan reaching 2.4% [32].

In the primary dentition, the teeth that are most affected by agenesis are the upper lateral incisors followed by the lower central and lateral incisors [32], and no gender-related differences in prevalence are reported in agenesis in deciduous dentition [20]. In case of a lack of elements of the deciduous series, the agenesis of the corresponding tooth of the permanent series is often associated [36].

In the case of permanent teeth and excluding third molars, the incidence of agenesis ranges from 2.6% to 11.3% depending on the different demographic and geographical profiles [32]. In non-syndromic patients with agenesis, 83% of patients have one or two missing permanent teeth, while only 0.14% present with oligodontia [37].

Evidence from the literature indicates that dental agenesis does not show a statistically significant preference for either the maxilla or the mandible. Nonetheless, the anterior region is more commonly affected [35,37], and unilateral agenesis was found to be more frequent than bilateral [32]. Multiple studies conducted on both Caucasian populations and black populations have shown a higher prevalence of dental agenesis in females compared to males [22,32,35,37].

These findings primarily refer to agenesis excluding third molars. However, even when third molars are considered, the same trend persists, with a higher percentage of third molar agenesis also reported in females [32]. Though this pattern appears to be consistent, it has been suggested that the apparent higher prevalence of dental agenesis in females may be influenced by sampling bias, with females being overrepresented in studies based on orthodontic records but not in those involving randomly selected populations [38].

Regarding the most affected teeth across several studies, it has been shown that the mandibular second premolar is the most frequently affected tooth, followed by the maxillary lateral incisor and the maxillary second premolar in Caucasian populations in North America, Australia, and Europe [37], with similar findings in studies evaluating additional populations [27]. Studies on orthodontic patients may report a slightly higher prevalence (about 1%) compared to the general population [27].

## 5. Patterns of Agenesis

There is evidence that distribution patterns may vary when considering populations with specific malocclusions: The prevalence of permanent tooth agenesis was more than twice as high in the Class II division 2 group compared to the control group of orthodontic patients [39], and similar findings were observed in Japan [40]. In both studies, the most commonly missing teeth of the Class II division 2 groups were the mandibular second premolars.

In contrast, a study conducted in Nepal found no significant difference in hypodontia among different classes of malocclusion [28].

Analysis of occlusal traits suggests a correlation between the number of missing teeth and specific morphological changes, including a progressive reduction in overjet, an increased interincisal angle, and shortened upper and lower dental arch lengths [29]. Furthermore, when compared to individuals without agenesis, patients with non-syndromic oligodontia exhibited significant distal angulation and delayed developmental stages of the mandibular second premolars [25].

Other dental characteristics and occlusal features commonly associated with the absence of maxillary lateral incisors include delayed dental development, microdontia of the contralateral maxillary lateral incisor, and palatal displacement of maxillary canines [35].

In recent decades, clinicians have reported a marked increase in the prevalence of tooth agenesis, suggesting a potential shift in epidemiological patterns or improved diagnostic detection [20,32].

## 6. Etiopathogenesis

Tooth agenesis can be associated with malformation processes of syndromic nature or occur in healthy subjects. A clear distinction based on gene classification is not possible because the same gene may be responsible for different forms of tooth agenesis, both syndromic and isolated. In a pivotal study that reviewed 393 clinical cases, 20 causative genes were identified, most notably *EDA, MSX1*, *WNT10A*, and *PAX9*, each showing distinct genotype–phenotype correlations [41]. Other minor genes responsible for both syndromic and isolated tooth agenesis are reported. If specific variants are found, the pattern and severity of missing teeth could be predicted as described in the following. Table 1 summarizes the major genes involved in syndromic and non-syndromic tooth agenesis.

***EDA*** **gene**

The tenth locus for tooth agenesis has been mapped on the X chromosome at Xq13.1 and corresponds to the *EDA* gene. Pathogenic variants in this gene are responsible for X-linked hypohidrotic ectodermal dysplasia (XLHED, OMIM #305100), making it a major contributor to ectodermal dysplasia in humans [42]. The *EDA1* isoform plays a crucial role in the development of sweat glands, teeth, and hair, whereas the function of isoform I remains unclear [43]. In a clinical study, individuals with *EDA* pathogenic variants presented variable phenotypes ranging from selective tooth agenesis and syndromic forms. Two heterozygotes showed mild phenotypes with no or only one missing tooth, while two homozygotes had similarly mild presentations. However, a third homozygous patient showed severe oligodontia with 15 missing permanent teeth. Across 23 cases involving *EDA* pathogenic variants, the average number of missing teeth was 12.6. The agenesis pattern predominantly involved anterior teeth, including mandibular lateral incisors (91.3%), mandibular central incisors (89.1%), and maxillary lateral incisors (84.8%).

***MSX1* gene**

The *MSX1* gene plays a pivotal role in craniofacial development and odontogenesis. It was the first gene identified in non-syndromic tooth agenesis in humans. Formerly known as homeobox7 (*HOX7*), *MSX1* is also associated with autosomal dominant syndromic conditions as well as autosomal dominant non-syndromic cleft lip (OMIM #608874), Wolf–Hirschhorn syndrome (OMIM #194190), autosomal dominant hypodontia (OMIM #106600), and Witkop syndrome (OMIM#189500) [44]. Evidence from transgenic mice homozygous for *Msx1* confirms the association with facial and dental abnormalities, including cleft palate, mimicking human phenotypes. *MSX1* encodes a transcriptional repressor involved in both the Wnt and BMP4 pathways. Pathogenic variants in this gene are typically associated with isolated tooth deficiencies. In Fournier’s study, clinical data show that 62.02% of patients with *MSX1* pathogenic variants presented with isolated tooth agenesis, 21.25% with oral clefts, 10% with Witkop syndrome, and 6.25% with Wolf–Hirschhorn syndrome [45,46].

***WNT10A* gene**

The *WNT* gene family is essential for the regulation of growth processes in orofacial tissues and dental development. Pathogenic variants in *WNT10A* are the most frequent genetic cause of isolated hypodontia and oligodontia, mostly with autosomal recessive inheritance. In animal models, inactivation of Wnt signaling results in facial underdevelopment, cleft lip or palate, and reduced tooth size due to diminished mesenchymal expression during tooth bud formation [47]. Biallelic pathogenic variants are generally associated with more severe phenotypes, including the absence of several permanent teeth, taurodontism, and abnormal root and crown morphology, and are also linked to odonto-onycho-dermal dysplasia (OMIM #257980) and Schöpf–Schultz–Passarge syndrome (OMIM #224750) [48]. In a Turkish family without consanguinity, a clear dosage effect was observed: The homozygous proband had only three permanent teeth, while the heterozygous parents each missed only two [41]. A broader study of 102 cases (74 isolated, 28 syndromic) confirmed *WNT10A* as the cause in 53.84% of syndromic forms, with an average of 13 missing teeth per patient. Maxillary second premolars were most frequently absent (82.4%), while maxillary central incisors were rarely affected (6.4%). Pathogenic variants in *WNT10B*, although sharing 59.2% amino acid sequence identity with *WNT10A*, resulted in a different pattern: The most often missing were mandibular lateral incisors (91.7%), with mandibular first premolars rarely affected (8.3%).

***PAX9* gene**

*PAX9* is the most common gene involved in autosomal dominant non-syndromic tooth agenesis and plays a crucial role in molar development [49]. Knockout mice lacking *Pax9* not only failed to develop teeth but also displayed abnormal development of organs, musculature, and skeletal structures, often leading to neonatal death [50]. In human studies, individuals with *PAX9* pathogenic variants were missing on average 11.7 teeth, with agenesis most prevalent in maxillary second molars (86.6%), mandibular second molars (86.6%), maxillary second premolars (70.6%), and maxillary first molars (69.1%), whereas maxillary central incisors were affected in only 6.7% of cases. Interestingly, affected individuals also reported reduced bitter taste perception, suggesting broader implications of *PAX9* in sensory development [41].

***Minor* genes**

Several additional genes contribute to distinct patterns of tooth agenesis.

In 16 cases with *LRP6* gene pathogenic variants, patients averaged 13 missing teeth, with maxillary lateral incisors frequently absent, while maxillary central incisors remained unaffected. In a family, *LRP6* caused agenesis of 15 permanent teeth and taurodontism, while in another family, a different variant resulted in 12 missing teeth [7].

*KREMEN1* pathogenic variants are associated with a symmetrical anterior agenesis pattern, with 15 patients missing on average 10 teeth, especially the maxillary and mandibular lateral incisors and mandibular central incisors [51].

In 11 cases involving *PITX2*, the phenotype was more severe, with an average of 16 missing teeth and frequent absence of maxillary central incisors, while mandibular first molars were consistently preserved [52].

*SMOC2* pathogenic variants led to a highly specific pattern: All six analyzed patients exhibited agenesis of both maxillary and mandibular second premolars, with an average of 10 missing teeth [53].

The *STHAG2* locus, first reported in 1998 in a large consanguineous Pakistani family, is associated with autosomal recessive hypodontia linked to a region on chromosome 16q12.1; affected individuals showed eruption failure and early tooth loss [54].

The *SHH* gene, a key regulator of craniofacial patterning, is associated with cleft lip, defective tooth morphology, and abnormal jaw development when mutated; heterozygous variants are associated with single median maxillary central incisor (OMIM #147250) [55]. Similarly, the TGF family—including *BMP2* and *BMP4*—is critical in odontogenesis; disruptions in these pathways lead to altered tooth size, crown malformations, and molar agenesis [56]. *GREM2* encodes a member of the DAN family of BMP (bone morphogenetic protein) antagonists. This gene plays a regulatory role in embryonic development, particularly in the morphogenesis of ectodermal organs such as teeth [57]. It works by inhibiting BMP signaling, a pathway crucial for tooth initiation, morphogenesis, and differentiation. During normal odontogenesis (tooth development), BMPs promote epithelial–mesenchymal interactions that drive the formation of dental structures. GREM2 acts as an antagonist of bone morphogenetic proteins (BMPs), playing a critical role in regulating signaling pathways during tooth development. By suppressing BMP activity, GREM2 ensures balanced morphogenetic signals necessary for normal odontogenesis. When *GREM2* is mutated or dysregulated, this signaling balance is disrupted, often resulting in defective tooth formation or oligodontia, a condition characterized by the congenital absence of six or more permanent teeth [57].

In addition to *GREM2*, variants in the *TSPEAR* (thrombospondin-type laminin G domain and epilepsy-associated repeats) gene have been implicated in tooth agenesis. Pathogenic variants in *TSPEAR* have been associated with autosomal recessive non-syndromic oligodontia. Notably, novel *TSPEAR* variants were identified in a Korean family with isolated oligodontia [58] and have also been observed in broader population studies [59].

## 7. Clinical Aspects

From a clinical perspective, it is of particular interest to identify whether specific phenotypic features are characteristic of patients with tooth agenesis. Among the studies addressing this issue, there is one that investigated the association between patterns of congenitally missing teeth and ectodermal clinical manifestations in patients with oligodontia [60]. The study sample included patients with isolated oligodontia (oligodontia/I), syndromic oligodontia (oligodontia/S), and a control group of individuals with complete permanent dentition. The main extra-oral symptoms observed in patients with oligodontia/S included dry skin, sparse hair, lessened or increased eyebrows, abnormal nails, and reduced sweat and tear secretion. When comparing the control group with patients with isolated oligodontia (oligodontia/I), the latter were found to have a deep-set plica mentalis, increased interocclusal distance, and an altered maxilla–mandible relationship. Additionally, dry skin was more frequently reported in the oligodontia/I group than in the control group.

**Figure 1:**
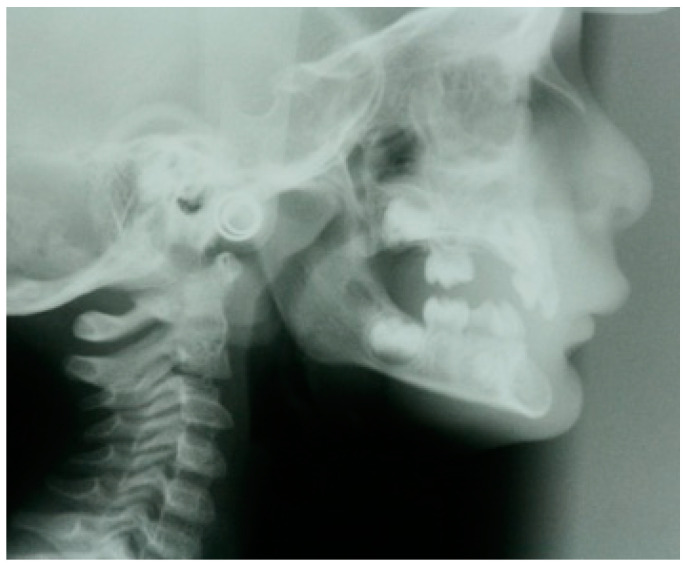
X-ray in a child with oligodontia. Note the characteristic chin projection.

Another study, conducted on a sample of 259 French patients aged between 12 and 18 years, identified a correlation between tooth agenesis and specific cephalometric parameters, such as transverse maxillary constriction, facial divergence, and anterior projection of the chin symphysis [61]. In our experience, we agree with these observations, having observed the presence of these clinical signs in patients with non-syndromic oligodontia (Figure 1).

Furthermore, a case report by Sasaki and colleagues [62] followed the craniofacial growth of a Japanese child with oligodontia associated with ectodermal dysplasia. The patient received prosthetic treatment with partial dentures in both jaws. Mandibular movement, dental casts, and both lateral and frontal cephalometric radiographs were assessed at the initial visit (at 7 years and 8 months of age) and again 1 year and 8 months later. An improvement in the stability of mandibular movements was observed between the two evaluations, both with and without partial dentures. Moreover, the mandibular length was found to be greater than the average for Japanese children of the same age. These findings suggest that functional development of mandibular movements may positively contribute to craniofacial growth in patients with oligodontia.

## 8. Therapy

Different treatment options are available for dental agenesis, ranging from orthodontic alone to combined orthodontic, surgical, and implant-based approaches, depending on the severity of the condition [63]. Specifically, the therapeutic approach may vary depending on whether the patient presents hypodontia (absence of a single or a few teeth) or oligodontia (absence of more than six teeth). One of the most commonly missing teeth is the maxillary lateral incisors. In this context, Schroeder and coauthors conducted an analysis of the advantages and disadvantages of treatment with or without substitution of canine in the place of second incisor [63]. Several factors play a critical role in treatment planning, including the patient’s age, smile analysis, and the morphology and color of the canines. In younger patients, space closure is often recommended, as the alternative approach of maintaining or creating space for future implants is associated with a risk of alveolar bone resorption in both height and thickness over time. In cases where patients with lateral incisor agenesis also display high gingival exposure while smiling, there is an increased risk that the lack of interdental papillae, gingival discoloration, and progressive bone loss may negatively affect the aesthetic and functional outcomes of an implant-based or orthodontic treatment plan. Conversely, implant therapy may be more appropriate in adult patients with low smile arch and no gingival display, minimizing these risks.

When opting for space closure, the authors recommend reshaping the canines to better resemble lateral incisors both aesthetically and functionally. In addition, canine coloration should be harmonized with adjacent teeth to improve the overall aesthetic result. For both treatment modalities—closing spaces and opening spaces—post-orthodontic retention is strongly recommended to maintain the results achieved.

The systematic review by Andrade and coauthors [64] evaluated the efficacy and safety of three treatment modalities for maxillary lateral incisor agenesis: space closure with canine reshaping to replace the lateral incisors, space opening with placement of a fixed prosthetic bridge, and space opening with placement of a single-unit implant-supported crown. The authors concluded that there is currently no strong scientific evidence to recommend or discourage any particular treatment as superior for this condition. Nonetheless, early diagnosis of hypodontia is considered crucial to enable timely planning of a multidisciplinary treatment approach, thereby increasing the chances of achieving both functional and aesthetic outcomes in the long term [12,65].

The management of multiple agenesis cases is undoubtedly more complex. However, the literature does include case reports [66,67]. The treatments described are established on an individual basis but have in common the management of spaces and the restoration of normal function by overcoming the absence of teeth with agenesis.

The article by Ephraim and coauthors describes a 10-year-old child presenting with complete agenesis of more than 10 deciduous and permanent teeth, leading to significant speech difficulties due to the absence of multiple teeth [68]. Treatment included maxillary expansion and restorative procedures with removable partial dentures. This approach improved aesthetics, replaced the missing teeth, enhanced speech, and helped prevent future downward growth of the maxilla.

In the case of adult patients, such as the one described by Dhanrajani and colleagues [69], orthodontic therapy to optimize spaces is aimed at positioning removable and fixed prostheses. The implant solution is adopted if the conditions are favorable.

Another notable case in the literature is that of an 18-year-old patient with non-syndromic agenesis of seven permanent teeth, followed over a six-year period [69]. The treatment plan prioritized the preservation of existing deciduous teeth in the arch, followed by a combination of orthodontic and restorative interventions. Once occlusal stabilization was achieved, dynamic orthodontic treatment was initiated. Resin-based restorations were performed on the upper deciduous incisors and canines, as well as the mandibular second molars. Orthodontic retention was then provided. At the six-year follow-up, the patient exhibited a stable and functional occlusion.

These studies highlight the necessity of a multidisciplinary and case-specific approach in the management of complex cases of multiple dental agenesis. The collaboration of several specialists offers the advantage of better understanding the causes of the existing problem, researching the genetic causes, and evaluating the possibility that they can generate pathologies in other areas of the body. The aspects most closely linked to dental rehabilitation must be discussed and treated by specialists from the different branches to obtain the best possible result [70].

**Figure 2:**
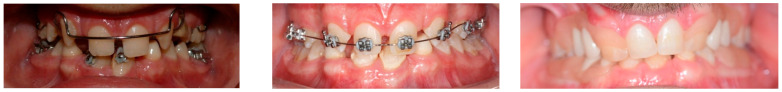
Images of teeth in a patient with oligodontia. From left to right are represented several steps of treatment.

An example of the treatment sequence—including orthodontic plates, fixed appliances, and subsequent prosthetic rehabilitation—is illustrated in Figure 2.

## 9. Conclusions

Tooth agenesis is a rare condition with different numbers and sites of affected teeth. Its origin is basically genetic. However, knowledge of the genes responsible for agenesis is not yet sufficient to clarify all aspects of pathogenesis, especially if this phenomenon occurs in an isolated (non-syndromic) form. Therapy depends on the presentation of the clinical condition and the age of the patient. The problems to be addressed are functional, aesthetic, and, in the case of young patients, also related to facial growth. All the solutions adopted involve the multidisciplinary involvement of the different branches of dentistry. The best condition for treating patients with agenesis, especially if numerous, is early diagnosis and following the entire process of facial development and occlusion of the patient with different therapeutic phases.

## Figures and Tables

**Table 1:** Main genes involved in syndromic (with extra-dental involvement) and non-syndromic (only dental involvement) tooth agenesis.

Gene	Function	Dental Involvement (Non-Syndromic Forms)	Extra-Dental Involvement (Syndromic Forms)
*EDA*	Ectodysplasin A	Tooth agenesis, selective, X-linked	X-linked hypohidrotic ectodermal dysplasia (XLHED)
*MSX1*	MSH Homeobox 1	Tooth agenesis, selective 1, with or without orofacial cleft (AD)	Ectodermal dysplasia A (AD), Witkop type (AD), orofacial cleft (AD)
*WNT10A*	Wingless type MMTV integration site family, member 10A	Tooth agenesis, selective (AD and AR)	Ectodermal dysplasia 16 (odontoonychodermal dysplasia) (AR); Schopf–Schulz–Passarge syndrome (AR)
*PAX9*	Paired box gene 9	Tooth agenesis, selective, 3 (AD)	Not reported
*LRP6*	Low-density lipoprotein receptor-related protein 6	Tooth agenesis, selective (AD)	Coronary artery disease, autosomal dominant (AD)
*KREMEN1*	Kringle domain-containing transmembrane protein 1	Ectodermal dysplasia 13, hair/tooth type (AR)	Ectodermal dysplasia 13, hair/tooth type (AR)
*PITX2*	Paired-like homeodomain transcription factor 2	Variation in tooth dimensions	Anterior segment dysgenesis (AD); Axenfeld–Rieger syndrome, type 1 (AD); ring dermoid of cornea (AD)
*SMOC2*	Sparc-related modular calcium-binding protein 2	Dentin dysplasia, type I, with microdontia and misshapen teeth (AR)	Not reported
*SHH*	Sonic Hedgehog signaling molecule	Not reported	Holoprosencephaly; microphthalmia/coloboma 5; single median maxillary central incisor (all AD)
*GREM2*	Gremlin 2, DAN family BMP antagonist	Tooth agenesis, selective, 9 (AD)	Not reported
*TSPEAR*	Tooth agenesis, selective, 10	Non-syndromic oligodontia	Ectodertamal dysplasia and tooth agenesis

AD = autosomal dominant; AR = autosomal recessive.

## Data Availability

Data discussed in this paper are contained within this article.

## Abbreviations

The following abbreviations are used in this manuscript:

ED	Ectodermal dysplasia
NMT	Number of missing teeth
TMD	Temporomandibular disorders
AD	Congenital dental agenesis
PP	Ponticulus Posticus
AOL	Atlantooccipital Ligament
